# Genomic insights into zoonotic transmission and antimicrobial resistance in *Campylobacter jejuni* from farm to fork: a one health perspective

**DOI:** 10.1186/s13099-022-00517-w

**Published:** 2022-12-05

**Authors:** Yara El dessouky, Salma W. Elsayed, Nehal Adel Abdelsalam, Nehal A. Saif, Avelino Álvarez-Ordóñez, Mohamed Elhadidy

**Affiliations:** 1 Biomedical Sciences Program, University of Science and Technology, Zewail City of Science and Technology, Giza, Egypt; 2 Center for Genomics, Helmy Institute for Medical Sciences, Zewail City of Science and Technology, Giza, Egypt; 3 Department of Microbiology and Immunology, Faculty of Pharmacy, Ain Shams University, Cairo, Egypt; 4 Department of Microbiology and Immunology, Faculty of Pharmacy, Cairo University, Cairo, Egypt; 5 Department of Food Hygiene and Technology and Institute of Food Science and Technology, Universidad de León, León, Spain; 6 Department of Bacteriology, Mycology and Immunology, Faculty of Veterinary Medicine, Mansoura University, Mansoura, Egypt

**Keywords:** Campylobacteriosis, *Campylobacter jejuni*, Whole genome sequencing, Molecular subtyping, Surveillance

## Abstract

**Background:**

Campylobacteriosis represents a global public health threat with various socio-economic impacts. Among different *Campylobacter* species, *Campylobacter jejuni* (*C. jejuni*) is considered to be the foremost *Campylobacter* species responsible for most of gastrointestinal-related infections. Although these species are reported to primarily inhabit birds, its high genetic and phenotypic diversity allowed their adaptation to other animal reservoirs and to the environment that may impact on human infection.

**Main body:**

A stringent and consistent surveillance program based on high resolution subtyping is crucial. Recently, different epidemiological investigations have implemented high-throughput sequencing technologies and analytical pipelines for higher resolution subtyping, accurate source attribution, and detection of antimicrobial resistance determinants among these species. In this review, we aim to present a comprehensive overview on the epidemiology, clinical presentation, antibiotic resistance, and transmission dynamics of *Campylobacter*, with specific focus on *C. jejuni*. This review also summarizes recent attempts of applying whole-genome sequencing (WGS) coupled with bioinformatic algorithms to identify and provide deeper insights into evolutionary and epidemiological dynamics of C. *jejuni* precisely along the farm-to-fork continuum.

**Conclusion:**

WGS is a valuable addition to traditional surveillance methods for *Campylobacter*. It enables accurate typing of this pathogen and allows tracking of its transmission sources. It is also advantageous for in silico characterization of antibiotic resistance and virulence determinants, and hence implementation of control measures for containment of infection.

## Introduction

The *Campylobacter* genus has approximately 57 species and some of these species are of clinical and veterinary relevance [1]. Among these species, thermophilic *Campylobacter* including *Campylobacter jejuni*, are the most common causative agents of campylobacteriosis. Other emerging *Campylobacter* species, such as *C. sputorum*, *C. upsaliensis, C. ureolyticus, C. lari*, and *C. hyointestinalis* also contribute to a wide range of gastrointestinal diseases [2]. *Campylobacter* is a Gram-negative bacillus with a characteristic spiral shape and polar flagella that propel the cells in a corkscrew-like fashion [3]. They have an optimal growth between 37 and 42^o^ C [4]. *C. jejuni* colonizes the gastrointestinal tract of most warm-blooded animals as commensals [5]. Chickens are reported to be one of the main sources of infection to humans. However, recent reports have also highlighted the role of wildlife and the environment (e.g. soil and water) in disease transmission [6]. The prevalence of *Campylobacter* infections is a critical global health concern. The World Health Organization (WHO) declared that *Campylobacter* species are responsible for 96 million cases of enteric infection worldwide [7]. The European Union (EU) tagged campylobacteriosis as the most reported zoonotic infection in 2020, responsible for over 60% of all documented cases [8]. The clinical presentations of *C. jejuni-*mediated infection vary from self-limiting diarrhea and abdominal pain to more serious extraintestinal infections [9].

Several molecular subtyping approaches (i.e*.,* amplicon-based typing, sequence-based typing, and restriction-based typing) have been implemented to investigate the epidemiology of *C. jejuni* [10]. However, systematic surveillance and epidemiological studies of *C. jejuni* are burdensome because of the sporadic nature of *Campylobacter* infections and the low discriminatory resolution of the traditional subtyping methods [10]. Thus, there is a demanding need for developing rapid subtyping methods with higher discrimination to track outbreak-causing lineages, predict antimicrobial resistance, determine accurate source attribution, and identify transmission dynamics. Whole genome sequencing (WGS) is a cutting-edge analytical method that enables reliable identification and characterization of foodborne pathogens. It can be used to tackle the challenges of the traditional molecular subtyping approaches. For example, WGS enabled the representation of global *Campylobacter* isolates and provided new means to detect disease-causing variants and host-related risks [11]. WGS opened new frontiers to explore the epidemiology of *C. jejuni* in populations, its capabilities for host adaptation, and its transmission from animal reservoirs to humans. Moreover, in the light of WGS, antimicrobial resistance (AMR) determinants can be predicted taking into consideration their composite transmission dynamics [12].

In this review, we highlight the impact of campylobacteriosis on human health, transmission of *C. jejuni* from animals to humans, genomic plasticity, and relevant information on epidemiological surveillance and antibiotic resistance. We will compare the performance of traditional techniques with that of whole genome sequencing in addressing different epidemiological questions related to *C. jejuni*.

### Impact of *campylobacter *on human health

Compared to other gastrointestinal pathogens*, Campylobacter* is highly infectious with an infective dose of 500 to 800 organisms for *C. jejuni* [13]. Although most of the infections caused by *C. jejuni* to humans are observed as isolated cases, outbreaks can take place [14]. *Campylobacter* infection varies from a self-limiting disease to serious extraintestinal infection. The most frequent clinical symptoms of campylobacteriosis are unspecific including fever, abdominal cramps, general malaise, muscle pain, diarrhea, and acute uncomplicated enterocolitis. Chronic gastrointestinal complications of *Campylobacter* infection include irritable bowel syndrome (IBS), inflammatory bowel disease, functional dyspepsia, and colitis [15]. While the disease is typically mild in healthy adults, severe and extended course of the disease, including bacteriemia, is a potential threat to young children, elderly adults, and immunocompromised patients [16].

*Campylobacter* infection and post-infection complications are quite rare. Nevertheless, many of these complications have a worse prognosis than the acute disease itself. The intensity of symptoms is thought to be affected by co-infection with another foodborne bacteria [17]. The most important extraintestinal complications are bacteremia, meningitis, hepatitis, endocarditis, and pulmonary infection [9]. The evolution of *Campylobacter* infections to a critical systemic illness, resulting in sepsis and death, is remarkably uncommon with a case-fatality rate of 0.05 in every 1000 infections [18]. Guillain–Barre syndrome (GBS) is a rare autoimmune neurological disorder in which peripheral nerves are demyelinated [19]. *C. jejuni* infection is the most common preceding infection and was reported in about 30% of GBS cases [19]. Specific *C. jejuni* serotypes have been associated with an increased risk of GBS (capsular types HS19, HS2, HS41, HS1/44c, HS4c, HS23/36c) [19]. Not all patients with cross-reactive antibodies develop neurologic manifestations. This can be explained by host determinants of post-*Campylobacter* GBS, particularly human lymphocyte antigen type [20]. The clinical isolate 81–176 of *C. jejuni* may be involved in development of colorectal cancer due to the cytolethal distending toxin [21].

### Zoonotic transmission of *Campylobacter jejuni*: one species, different hosts

*Campylobacter* is primarily a zoonotic disease-causing bacterium [13]. Poultry are the main natural reservoirs, especially for *C. jejuni*, with a cecal content of up to 1 × 10^8^ CFU/g [22]. By contaminating the carcass and surviving processing in slaughterhouses, *C. jejuni* can be transferred to humans via undercooked chicken meat or through cross-contamination of other foodstuffs at the kitchen [23]. Contaminated water, milk, and dairy products are other sources of infection [24]. In addition, *C. jejuni* and other *Campylobacter* species are frequently found in other animal reservoirs, in up to 90% of cattle, 85% of pigs, and 17.5% of sheep and goats [9]. Cattle isolates are usually clustered in *C. jejuni* clonal complexes (CC) CC-21, CC-45, CC-48, CC-42, CC-61 and CC-206 [25].

Although cross contamination of food represents the most common source to contact *Campylobacter*, different reservoirs of human campylobacteriosis may potentially play a role in the epidemiology and transmission of *Campylobacter*. Other unconventional routes of transmission of *Campylobacter* spp. are surface water, pets, and wild birds [26]. Surface water accounts for a significant number of human cases. Contamination of surface water with wild animal feces and agricultural waste makes water a collection vessel of different *Campylobacter* strains from various hosts [26]. Pets were found to cause considerable number of human cases where the transmission of *Campylobacter* can be bi-directional from owners to pets and vice versa. Pets may acquire infection in parallel with their owners from a common source [26]. While a genomic characterization and profiling of *C. jejuni* showed a partial overlap between isolates from livestock, pets, and clinical cases, isolates from pets showed specific genomic profiles. Thus, pets can be a potential reservoir for *C. jejuni* [27]. Wild birds acquire *C. jejuni* from contaminated water, refuse dumps, and waste from animal farms, pets, and humans [28]. Their body temperatures, in addition to foraging and breeding habits, enable wild birds to be potential reservoirs and spreading routes of *Campylobacter* [28]. Some studies detected antibiotic resistant *C. jejuni* in wild birds in many geographical locations, which poses a concern in urban areas and agricultural farms with increased wild birds population [29]. Other studies showed that food and human *C. jejuni* isolates differed from those of wild birds [30]. Other environmental habitats such as soil are directly or indirectly implicated in human campylobacteriosis [31]. Intriguingly, *Campylobacter* can be also transmitted through flies to chicken flocks and possibly to humans [32].

Although it has strenuous growing conditions in the lab, *C. jejuni* acquired resistance to a plethora of stressors, such as low pH, temperature variability, oxidative stress, osmotic pressure, and antimicrobials [33]. These mechanisms enable *C. jejuni* to survive and transmit between diverse hosts [33]. Tolerance to environmental stressors is frequent among disease-causing lineages and can result in more adapted isolates, with an impact in the general epidemiology of *C. jejuni* [33]. Among the resistance mechanisms to environmental stressors are the transformation into a viable non culturable state (VBNC), biofilm formation, and mutations specific to certain lineages [33, 34]. Some of the host-generalist and host-specific strains of *C. jejuni* were proved to survive in aerobic conditions and under oxidative stress [35].

### Mechanisms underlying genomic plasticity and host adaptation

Pan-genome is a term used to describe the entire gene collection identified in a species. The term encompasses two classes: core genome and accessory genome [36]. The core genome represents the set of genes present in every isolate of the species and carries out the necessary cellular functions [36]. The accessory genome constitutes the variable dispensable genome acquired for adaptation and is present only in a few strains or even unique to one strain. Pan-genomic studies highlight the marked variations of bacterial genomes between different genera and species and even between different strains of the same species. These variations can be referred to as genomic plasticity [37].

Almost all bacterial genomes have mosaic structures that are assembled from different DNA segments during evolution and adaptation [38]. The exchange of DNA between bacterial cells occurs via horizontal gene transfer (HGT) either by conjugation, transformation, or transduction [38]. While plasmids and conjugative transposons mediate conjugation, phages that infect bacterial cells mediate transduction [38]. Transformation, on the other hand, occurs when naturally competent bacteria take up extracellular DNA from the environment [38]. The genome of *C. jejuni* is relatively small, however, it is characterized by high variation even at the strain level [31]. *C. jejuni* is naturally competent as it uses a DNA uptake system called type II secretion system to transport foreign extracellular DNA to the cytoplasm [5]. To integrate the homologous DNA into the chromosome, *C. jejuni* uses RecA recombinase, a protein that promotes homologous recombination [5].

A recent study elucidated the role of the chicken gut environment, particularly that of the ceca, in providing suitable conditions for recombination to occur [39]. The results suggested that increased HGT in chicken gut promotes the genetic diversity and hence the adaptability of *C. jejuni* to the constantly challenging gut environment [39]. The exchange of DNA between bacterial cells contributes to bacterial adaptation to a wide range of environmental conditions and to the colonization of multiple niches. Moreover, it plays an essential role in the evolution of antibiotic resistance and bacterial virulence [38]. When comparing the pattern of genetic variations observed in human pathogenic isolates to that of poultry isolates belonging to the same clonal complex, evidence is further supporting that host-specific mutations develop within certain hosts [5]. Although host specialists are mainly found in only one host species while host generalists are commonly associated with multiple hosts, host specialists have been recently shown to infect more than one definite host [40, 41]. This host adaptation was recently supported by Nennig et al. by implementing different typing schemes based on WGS gene-by-gene approach. They concluded that some *C. jejuni* lineages have clonally expanded and can colonize or infect multiple hosts as they show adaptation to different niches [42]. Remarkably, a recent study has found that host generalist lineages are better equipped to withstand hostile environmental conditions compared to host specialists, but this needs to be further characterized at the molecular level [33]. To provide better insights into the emergence of generalists, Woodcock et al. investigated the role of genomic plasticity in the coexistence of generalist and specialist *Campylobacter* lineages. They concluded that the ecological generalism observed in some *C. jejuni* isolates reflected their genotypic and phenotypic plasticity and resulted in their rapid host adaptation in different host environments [37].

### WGS and *Campylobacter jejuni* genomic diversity

As mentioned in the previous section, certain lineages of *C.* *jejuni* are specific to a particular host species that is related to host adaptation [40–43]. Another interesting example is the recent study conducted by Parker et al. where two strains of *C. jejuni* colonizing guinea pigs were compared with well characterized *Campylobacter* strains [44]. They found that isolates from guinea pigs were of novel sequence type, distinct from other known *Campylobacter* strains, and had genes gain and loss in their genomes. This can further support that genomic divergence occurs as a result of host adaptation mechanisms [44]. This extensive genome variability may play an important role in *C. jejuni* survival and host adaptation [31].

One application for WGS is studying bacterial genomic diversity accrued by animal colonization and human infection [45–48]. Golz et al. identified hybrid strains of *C. jejuni* where extensive gene transfer between the two species interfered with the analysis of species differentiation and multilocus sequence typing (MLST) [49]. These adaptation mechanisms lead to the emergence of host-associated genes or clusters of genes that can be resolved by WGS, which can be of a remarkable use to detect and infer host adaptation mechanisms in *C. jejuni* [50].

### Epidemiological surveillance

Systematic surveillance of *C. jejuni* infection is a complex process due to high genomic diversity of the bacteria and interactions between different routes of transmission [31, 43]. In the context of epidemiological surveillance of *C. jejuni,* bacterial subtyping is crucial to differentiate bacteria sharing certain genomic similarities and link them to the same source [51]. The distinction between epidemiologically related incidents and sporadic cases requires high-resolution detection and typing techniques [10]. This is especially important to track both point source and diffuse outbreaks.

### Molecular typing schemes

Consistent detection and identification of *C. jejuni* genotypes is challenging due to their high variability. Additionally, traditional culture-based methods fail to detect bacterial variations [52]. They are time-consuming, low throughput, laborious, of low sensitivity, and may yield false negative results if the bacteria are in VBNC state [52]. Without accurate diagnostic tests to detect the presence of *Campylobacter*, precise differentiation and diagnosis of enteric illnesses caused by other bacteria including *Salmonella*, *Shigella*, and *Yersinia* can be challenging [53]. *Campylobacter* species, specifically *C. jejuni*, possess highly changeable physiology, metabolism, and phenotypic diversity. Consequently, traditional detection methods are inadequate, inaccurate, and not sensitive enough [52]. Thus, research is now driven towards devising more accurate, cost- and time-effective detection methods, especially in the food industry where screening is crucial to prevent transmission [54].

Molecular typing schemes have been previously used for *C. jejuni*, including in outbreak investigations, host-association, and population structure studies. Restriction fragment length polymorphism, ribotyping, PCR-based methods, pulsed-field gel electrophoresis, and antigen gene sequence typing (as for *flaA* and *porA* genes) are considered as robust and reproducible genotyping methods in understanding the biology of *C. jejuni*. These typing methods can be implemented for different epidemiological purposes, including for *Campylobacter* subtyping, phylogenetics, identification of outbreak-inducing lineages, and epidemiologic tracking [55]. Nonetheless, the aforementioned subtyping methods have limited discrimination capacity in epidemiological investigations and have several drawbacks such as poor discriminatory power and incompatibility with high throughput applications [55]. MLST has been previously employed over the past decades as the gold standard subtyping method in studying relationships between *Campylobacter spp*. strains, investigating the evolution, population structure, and the molecular epidemiology of the disease and exploring the potential host reservoirs and host associations [56]. MLST classifies isolates based on polymorphisms present in certain regions of housekeeping genes. Closely related sequence types are grouped under clonal complexes [57]. According to MLST genotyping, strong associations were found between *C. jejuni* host generalists and some clonal complexes (CC) including: ST-21, ST-48, ST-206, and ST-45 [41]. These lineages are causative sources of human diseases [58]. Despite broad geographical distinction between *Campylobacter* species, specific STs are found to be associated with infections in specific countries. For instance, ST-22 and ST-4526 were found in Finland and Japan, respectively, while ST-190 and ST-474 emerged in New Zealand [36]. The comparison of geographically distinctive *Campylobacter* isolates is made possible by molecular typing and WGS. One example is the analysis of *Campylobacter* genomes in UK and North America [59]. The analysis concluded the clustering of these isolates based on variations of highly recombining genes while the isolates were geographically distant [59]. Another study compared *C. jejuni* isolates in Egypt and UK. CC21 isolates from the same country shared more accessory genome genes that were lineage-specific; thus, isolates were geographically clustered [60]. Therefore, biogeographical identification of signatures from *Campylobacter* genomes can help improve campylobacteriosis source attribution and implement reliable intervention strategies.

While MLST is superior to other classic typing methods in studying the population structure for source attribution and the identification of transmission routes in outbreaks, it has several drawbacks [57]. MLST alone may not be sufficient to resolve closely related bacterial strains and, in this case, a specific MLST scheme should be devised [57]. Therefore, new tools and screening techniques were needed for epidemiological surveillance of *C. jejuni* to address the limitations of the classic typing methods.

### WGS in the surveillance of *Campylobacter*

WGS technologies are continuously evolving to sequence nucleotides at reasonable speed and low cost. As a result, more and more bacterial genomes are becoming available for analysis and routine surveillance and outbreak tracking are becoming more feasible [61]. Instead of conventional genotyping, which is restricted to only some parts of the genome, WGS provides information on the entire genomic content of isolates. WGS can thus enhance microbial safety surveillance to help control foodborne outbreaks [61]. WGS is characterized by enhanced discriminatory power at the strain level, also enabling the association of specific genotypes with phenotypes that are clinically and epidemiologically relevant [62]. WGS based subtyping demonstrates several advantages over traditional genotyping methods, including in silico prediction of antimicrobial resistance determinants, attribution of transmission sources and routes, and enhanced surveillance of food-borne pathogens [63–65]. Therefore, WGS serves as an effective measure for controlling and preventing foodborne infections [62]. This is especially demonstrated during infectious disease outbreaks where WGS was used for typing [62]. WGS paves the way to characterize the genomic diversity among *Campylobacter* isolates; thus, improving decision making and intervention to control outbreaks [10].

### WGS and *C. jejuni* epidemiology

#### WGS-based subtyping

WGS analysis can be applied in real time to investigate epidemiologically-linked campylobacteriosis cases showing high similarities at the genomic level [66]. Coupling de novo assembly of genomes with a gene-by-gene analysis can expand from the classic MLST scheme to the core genome MLST scheme [11] (cgMLST), based on the analysis of a large number of genes shared by most of the members of a given bacterial group. cgMLST typing is routinely used to align *C. jejuni* genes for the identification of clonal complexes and has greater discriminatory power than conventional MLST, thus aiding in providing better insights into the origin of human campylobacteriosis cases [11].

A study by Cody et al. performed the first real-time genomic epidemiological investigation using a hierarchical whole genome MLST approach [67]. Over 1000 loci were extracted using a BIGSdb Genome Comparator in PubMLST. These loci were compared against 1643 publicly listed loci and complemented with a whole genome MLST analysis. The analysis aimed to identify diversity within the detected clusters to allow for the identification of temporal links between clusters in seemingly epidemiologically unrelated cases [67]. Further support to these findings was a study by Fernandes et al. where comparison of *C. jejuni* isolates against a reference nonrelated population showed that most of the apparently sporadic cases belonged to a cluster with fewer than 8 allele dissimilarities out of 1577 shared loci [68]. Another study used reference-based core-genome MLST analysis to examine a chicken-associated outbreak in Australia over 1271 loci, and found no more than one allele difference between the clinical isolates [69]. A study on the Walkerton outbreak in Canada indicated that four isolates were related on the clonal level and of limited variation on the genomic level. The isolates were different from one another by 15 single nucleotide variations and approximately 4 allele differences in a core genome scheme over 732 core loci [70].

#### WGS based source attribution

Source attribution of zoonotic diseases is defined as the assignment of the human clinical cases of infection to their reservoirs and transmission routes [71]. Different source attribution methods have been developed for foodborne pathogens. These methods can be defined as either top-down or bottom-up approaches. Top–down approaches assign human cases to the sources of infection and aid in predicting the risk of food production animals and other sources for causing infections in humans, advancing intervention strategies and public health in general [71–74]. The data for these methods can be provided by epidemiological methods, microbiological subtyping based methods, or both together [71]. On the other hand, bottom–up approaches predict the number of human cases caused by each source by first analyzing the contamination level and then moving upwards through the transmission chain [71]. The genetic analysis of foodborne pathogens plays a pivotal role in source attribution. In terms of foodborne pathogens, population structure defines the systematic differences in allele and phenotype frequencies in populations and subpopulations of a pathogen [75]. Consequently, probable risk factors and relative contribution of different sources can be determined. While source attribution depends on the accurate estimation of the frequency of different subtypes in each host reservoir, it may be challenging for some organisms such as *Campylobacter* to find specific host associated markers as the population is not properly structured into differentiated clusters [76, 77].

One approach to study population genetics is microbial genotyping of isolates from both human cases and possible sources in the food chain [77]. This approach depends on the bacteria being adapted to different hosts or ecological niches which leads to uneven distribution of sequence subtypes among host reservoirs [77]. These genomic signatures would help to understand *C. jejuni* evolution and track sources of human infection [76]. For proper source attribution, a typing method should be standard and valid to help reliable knowledge transfer among laboratories working on the analysis [71]. Moreover, the method should also be automated with a reference data set allowing for the establishment of nomenclature within the microbial species [71].

To apply WGS in source attribution, there have been several successful attempts to develop algorithms that provide optimal discriminatory power and proper modeling [73]. Recently, allelic variation has been analyzed using 15 host-segregating marker loci (including seven core genes, seven soft-core genes, and one accessory gene) derived from the pan-genome of *C. jejuni* reference strains [73]. These loci have been used in source attribution analysis as they retain high accuracy of attribution even between host specialist and generalist genotypes [73, 78, 79]. Six of the host-segregating loci encode hypothetical proteins and the remaining loci are involved in metabolic activities, signal transduction, protein modification, and stress response [73]. This typing method was reported to be of higher accuracy and segregation power than MLST [78]. It is advisable to use more than one molecular typing method for the investigation of *Campylobacter* populations [80].

Source attribution based on microbial subtyping can be classified according to the computational modeling used. The model-based molecular attribution can be applied to assess interventions used to halt disease transmission from farms to retail outlets to final human consumption (farm-to-fork) [74]. The differences in genotype frequency between various populations enables probabilistic assignment of isolates to populations [77]. Models can be frequency-matching models or population genetics models [71]. In frequency-matching models, subtype frequencies are compared and weighted assuming that subtypes are stable from their sources [71]. The population genetics models are probabilistic, and the parameters are assumed to be unknown [71]. The comparison of the genomic data available for strains may infer the link between strains from human and different sources. Examples of current population genetics models available are the STRUCTURE model and the Asymmetric Island Model [71]. STRUCTURE is a model-based clustering method designed to infer population structure and assign individuals to populations using genotype data [81]. STRUCTURE estimates genotype frequencies in each host species based on all the isolates. It estimates the population of origin for isolates of unknown origin [81]. The principle of this model is to estimate the allelic frequencies in different populations and their admixtures using Bayesian approach [81]. Tracing the sources of human cases is a use case of this model without admixture of the source strains. The strains should belong only to one of each population and each population should be of a specific source [71].

#### Antimicrobial resistance in *Campylobacter*

Although campylobacteriosis is typically self-limiting, with a short-duration, and rarely requires antimicrobial therapy, high-risk patients may receive an early antibiotic intervention to avoid serious complications [82]. Macrolides are the antibiotics of choice when treating *C. jejuni* infections and fluoroquinolones are used as an alternative therapy [83]. Tetracyclines are another alternative treatment for campylobacteriosis but not commonly used in clinical practice. Severe systemic campylobacteriosis may be treated with intravenous aminoglycosides [84]. *C. jejuni* has intrinsic resistance to a wide range of antibiotics including penicillin, most of the cephalosporins, vancomycin, cotrimoxazole, and rifampicin [83]. Moreover, a growing number of *Campylobacter* strains are developing resistance against quinolones and macrolides which are critically valuable antimicrobials in managing human infections [83]. In the past decades, the rise of antimicrobial resistance has become a significant global concern in both developed and developing countries. Resistance to antimicrobials is acquired and are mainly disseminated among *Campylobacter* strains via HGT and mutation-based mechanisms. Antibiotic-resistant strains are capable of modifying the antibiotic target sites, reducing cellular permeability to antibiotics, or hydrolyzing or effluxing antibiotic compounds [83].

#### Role of animals and food in transmission of antimicrobial resistance

The role of animals in the spread and transmission of AMR in humans is evident by studies that correlated the emergence and clonal expansion of resistant *C*. *jejuni* strains with the dissemination of resistance genes among various lineages as revealed by the association between different clones and antimicrobial resistance [85–88]. One of the main sources of AMR transmission from animal to human is the use of antibiotics in agriculture and veterinary fields. How the antibiotics are selected for use in these fields depends on the animal species itself, whether farming is commercial or domestic, and the availability of the antimicrobials under strict legalization work frame [89]. Multiple studies have detected AMR in *C. jejuni* not only in broiler products but also in livestock animals in different geographical locations suggesting their role as a probable source of clinically relevant antimicrobial-resistant *Campylobacter* spp. [90–92]. In an attempt to investigate the AMR genes transfer between bacterial isolates, a study explored the genomic determinants of AMR in *C. jejuni* isolated from humans, livestock, and sewage [93]. The results indicated the spread of some AMR determinants between *Campylobacter* species and the niches from which they are isolated. These study findings were in agreement with the results of resistome analysis obtained by Cobo-Díaz et al. [94]. A total of 39,798 publicly available *Campylobacter jejuni* genomes were studied, focusing on their sequence types and resistome profiles. These studies highlighted the association between the use of antimicrobial agents in veterinary settings, particularly poultry production, and the subsequent spread of AMR genes between *Campylobacter* isolates residing in humans, animals, and environment.

#### AMR and WGS

WGS analyses have served as a powerful tool for the accurate characterization and prediction of AMR within members of the *Campylobacter* genus [95–97]. In *Campylobacter*, antimicrobial resistance develops from either spontaneous mutations, acquisition of AMR genes, or both [83]. WGS is successfully applied to detect putative gene mutations that result in resistant phenotypes. It can also detect acquisition of DNA sequences associated with antibiotic resistance. The prediction can be further improved by verifying resistance markers and constructing a reliable pipeline. Several databases are available to detect AMR genes based on WGS technology such as ResFinder [98], Resfams [99], ARG-ANNOT [100], CARD [101], or NCBI AMRFinder [102]. Jointly with comparative genomic studies, the data obtained can unravel much about the unknown mechanisms of resistance and the role animals play in disseminating resistant strains in humans [95, 103].

#### Genome wide association studies and *Campylobacter*

Genome-wide association studies (GWAS) are increasingly being implemented in microbial genomics to statistically associate genetic elements with particular phenotypes [104]. With the cost effective availability of WGS, GWAS can be performed to identify the genetic components of any measurable heritable phenotype in a hypothesis-free manner [75]. Microbial GWAS analysis could reveal genes and mutations that are linked to antibiotic resistance, virulence, and host tropism [75]. GWAS is an example of a top-down approach because the genomic content of test and control groups is compared and analyzed to identify genetic variation that is associated with a specific trait. Bacteria are characterized by unique population genetics that impose challenges in applying microbial GWAS analyses [75]. Among these challenges are the genetic content and its high diversity. Early GWAS depended on expensive genotyping chips with known DNA probes which became obsolete by time due to the plasticity of bacterial genomes. WGS is a cheaper and more comprehensive for production of full sequences fast and in high throughput [105].

Microbial GWAS analyses are divided into either phylogeny-based, non phylogeny-based, or can be a combination of both. Machine learning predictive models can also be applied [105]. GWAS that were applied to identify SNPs and k- mers in microbial genomes have identified mutations and genes associated with antibiotic resistance, cancer, virulence and host preference [75]. Among the tools used for microbial GWAS are Scoary, TreeWAS, bugwas, and PySEER [106–109]. *C. jejuni* population lineages are clustered into clonal complexes that share genetic elements. Not all of these genetic elements are correlated to particular phenotypes as some elements are passed through clonal descent and not associated with the phenotype of interest [104]. GWAS analysis of *C. jejuni* revealed the association of the *cj1377c* gene with survival where protein expressed by cj1377c gene is involved in *C. jejuni* respiration and formate metabolism [104]. Another study showed that the gain and loss of the *pan*BCD genes, encoding the vitamin B5 biosynthesis pathway, is associated with rapid host adaptation. On one hand, vitamin B5 is present in cereals and grains, which are part of the chicken diet. On the other hand, it is found in a very low concentration in grasses on which cattle feed. The *pan*BCD genes were found almost globally in cattle isolates as *Campylobacter* needs to produce the vitamin to persist in cattle. Thus, host generalism in *Campylobacter* lineages linked to agricultural niches is probable as *pan*BCD genes persist in some isolates in chickens [110]. GWAS on *C.jejuni* isolates distinguished 28 genes that are significantly associated with highly prevalent and clinically related *C. jejuni* subtypes. Those genes are associated with iron acquisition, vitamin B5 biosynthesis, catalysis, and transport [111]. WGS together with GWAS could reveal novel source attribution markers that differentiated *C. jejuni* isolates from UK and France [74]. GWAS helped determining marker genes, where the absence/presence or mutations were associated with the adaptation of certain lineages of *C. jejuni* to specific host niches [112].

## Conclusion

The review summaries the WGS applications in the post genomic era to understand *C. jejuni* adaptation, antimicrobial resistance determinants, and transmission dynamics along the farm-to-fork continuum. The increasing use of WGS for epidemiological purposes can contribute to improve current surveillance programs. WGS provides high discriminatory resolution in comparison with traditional subtyping methods and will gradually replace these methods in surveillance studies. It should allow a more accurate identification of possible case clusters and resistome patterns to control and prevent more cases of campylobacteriosis. WGS can drive “One Health” epidemiological investigations by providing an unprecedented level of data that can be used to describe emerging trends. It can guide the establishment of links between animal and human health and the environment and clarify the direct or indirect role of *Campylobacter* ecology in its transmission to humans.

## Data Availability

Not applicable.

## Abbreviations

AMR: Antimicrobial resistance
BIGSdb: Bacterial Isolate Genome Sequence Database (BIGSdb)
CC: Clonal complex
C. jejuni: Campylobacter jejuni
cgMLST: Core genome multi-locus sequence typing
EU: European Union
GBS: Guillain–Barre syndrome
GWAS: Genome-wide association studies
HGT: Horizontal gene transfer
IBS: Irritable bowel syndrome
MFS: Miller Fisher Syndrome
MLST: Multi-locus sequence typing
PCR: Polymerase chain reaction
QRDR: Quinolone resistance determining region
RPP: Ribosomal protection proteins
ST: Sequence type
VBNC: Viable non culturable state
WGS: Whole genome sequencing
WHO: World Health Organization

## References

[1] Parte AC, Sardà Carbasse J, Meier-Kolthoff JP, Reimer LC, Göker M (2020). List of Prokaryotic names with standing in Nomenclature (LPSN) moves to the DSMZ. Int J Syst Evol Microbiol.

[2] Igwaran A, Okoh AI (2019). Human campylobacteriosis: a public health concern of global importance. Heliyon.

[3] Cohen EJ, Nakane D, Kabata Y, Hendrixson DR, Nishizaka T, Beeby M (2020). *Campylobacter jejuni* motility integrates specialized cell shape, flagellar filament, and motor, to coordinate action of its opposed flagella. PLOS Pathog..

[4] Davis L, DiRita V (2008). Growth and laboratory maintenance of *Campylobacter jejuni*. Curr Protoc Microbiol.

[5] Burnham PM, Hendrixson DR (2018). *Campylobacter jejuni*: collective components promoting a successful enteric lifestyle. Nat Rev Microbiol.

[6] Enany S, Piccirillo A, Elhadidy M, Tryjanowski P (2021). Editorial: The role of environmental reservoirs in *Campylobacter*-mediated infection. Front Cell Infect Microbiol.

[7] Havelaar AH, Kirk MD, Torgerson PR, Gibb HJ, Hald T, Lake RJ (2015). World Health Organization global estimates and regional comparisons of the burden of foodborne disease in 2010. PLOS Med..

[8] Authority EFS EC Control for DP (2021). The european union one health 2020. EFSA J.

[9] Kaakoush NO, Castaño-Rodríguez N, Mitchell HM, Man SM (2015). Global epidemiology of *Campylobacter* infection. Clin Microbiol Rev.

[10] Llarena A-K, Taboada E, Rossi M (2017). Whole-genome sequencing in epidemiology of *Campylobacter jejuni* Infections. J Clin Microbiol.

[11] Sheppard SK, Jolley KA, Maiden MCJ (2012). A gene-by-gene approach to bacterial population genomics: whole genome MLST of *Campylobacter*. Genes (Basel).

[12] Mouftah SF, Cobo-Díaz JF, Álvarez-Ordóñez A, Elserafy M, Saif NA, Sadat A, El-Shibiny A, Elhadidy M (2021). High-throughput sequencing reveals genetic determinants associated with antibiotic resistance in *Campylobacter* spp. from farm-to-fork. PLoS One.

[13] Janssen R, Krogfelt KA, Cawthraw SA, van Pelt W, Wagenaar JA, Owen RJ (2008). Host-pathogen interactions in *Campylobacter* infections: the host perspective. Clin Microbiol Rev.

[14] Olson CK, Ethelberg S, van Pelt W, Tauxe R V. 2008 Epidemiology of Campylobacter jejuni infections in industrialized nations. In: *Campylobacter*. ASM Press: Washington, 163–89. https://onlinelibrary.wiley.com/doi/10.1128/9781555815554.ch9

[15] Riddle MS, Gutierrez RL, Verdu EF, Porter CK (2012). The chronic gastrointestinal consequences associated with *Campylobacter*. Curr Gastroenterol Rep.

[16] Barker CR, Painset A, Swift C, Jenkins C, Godbole G, Maiden MCJ (2020). Microevolution of *Campylobacter jejuni* during long-term infection in an immunocompromised host. Sci Rep.

[17] Wang G, He Y, Jin X, Zhou Y, Chen X, Zhao J, Zhang H, Chen W (2018). The effect of co-infection of food-borne pathogenic bacteria on the progression of Campylobacter jejuni infection in mice. Front Microbiol.

[18] Acheson D, Allos BM (2001). *Campylobacter jejuni* infections: update on emerging issues and trends. Clin Infect Dis.

[19] Nyati KK, Nyati R (2013). Role of *Campylobacter jejuni* infection in the pathogenesis of Guillain–Barré syndrome: an update. Biomed Res Int.

[20] McCarthy N, Giesecke J (2001). Incidence of Guillain-Barré syndrome following infection with *Campylobacter jejuni*. Am J Epidemiol.

[21] He Z, Gharaibeh RZ, Newsome RC, Pope JL, Dougherty MW, Tomkovich S (2019). *Campylobacter jejuni* promotes colorectal tumorigenesis through the action of cytolethal distending toxin. Gut.

[22] Rosenquist H, Sommer HM, Nielsen NL, Christensen BB (2006). The effect of slaughter operations on the contamination of chicken carcasses with thermotolerant *Campylobacter*. Int J Food Microbiol.

[23] Humphrey TJ, Martin KW, Slader J, Durham K (2001). *Campylobacter* spp. in the kitchen spread and persistence. J Appl Microbiol.

[24] The global view of campylobacteriosis: report of an expert consultation, Utrecht, Netherlands, 9–11 July 2012. World Health Organization, Food and Agriculture Organization of the United Nations and World Organisation for Animal Health. 2013. https://apps.who.int/iris/handle/10665/80751. Cited 13 Jul 2022

[25] An JU, Ho H, Kim J, Kim WH, Kim J, Lee S (2018). Dairy cattle, a potential reservoir of human campylobacteriosis: Epidemiological and molecular characterization of *Campylobacter jejuni* from cattle farms. Front Microbiol.

[26] Mughini-Gras L, Pijnacker R, Coipan C, Mulder AC, Fernandes Veludo A, de Rijk S (2021). Sources and transmission routes of campylobacteriosis: a combined analysis of genome and exposure data. J Infect.

[27] Thépault A, Rose V, Queguiner M, Chemaly M, Rivoal K (2020). Dogs and cats: reservoirs for highly diverse *Campylobacter jejuni* and a potential source of human exposure. Animals.

[28] Sen K, Berglund T, Patel N, Chhabra N, Ricci DM, Dutta S (2022). Genotypic analyses and antimicrobial resistance profiles of *Campylobacter jejuni* from crows (Corvidae) of United States and India reflect their respective local antibiotic burdens. J Appl Microbiol.

[29] Hald B, Skov MN, Nielsen EM, Rahbek C, Madsen JJ, Wainø M (2016). *Campylobacter jejuni* and *Campylobacter coli* in wild birds on Danish livestock farms. Acta Vet Scand.

[30] Griekspoor P, Colles FM, McCarthy ND, Hansbro PM, Ashhurst-Smith C, Olsen B (2013). Marked host specificity and lack of phylogeographic population structure of *Campylobacter jejuni* in wild birds. Mol Ecol.

[31] Bronowski C, James CE, Winstanley C (2014). Role of environmental survival in transmission of *Campylobacter jejuni*. FEMS Microbiol Lett.

[32] Evers EG, Blaak H, Hamidjaja RA, de Jonge R, Schets FM (2016). A QMRA for the transmission of ESBL-producing *Escherichia coli* and *Campylobacter* from poultry farms to humans through flies. Risk Anal.

[33] Mouftah SF, Cobo-Díaz JF, Álvarez-Ordóñez A, Mousa A, Calland JK, Pascoe B (2021). Stress resistance associated with multi-host transmission and enhanced biofilm formation at 42 °C among hyper-aerotolerant generalist *Campylobacter jejuni*. Food Microbiol.

[34] Jackson DN, Davis B, Tirado SM, Duggal M, van Frankenhuyzen JK, Deaville D (2009). Survival mechanisms and culturability of *Campylobacter jejuni* under stress conditions. Antonie Van Leeuwenhoek.

[35] Nennig M, Clément A, Longueval E, Bernardi T, Ragimbeau C, Tresse O (2022). Metaphenotypes associated with recurrent genomic lineages of *Campylobacter jejuni* responsible for human infections in Luxembourg. Front Microbiol.

[36] Epping L, Antão E-M, Semmler T (2021). Population biology and comparative genomics of *Campylobacter* species. Curr Top Microbiol Immunol.

[37] Woodcock DJ, Krusche P, Strachan NJC, Forbes KJ, Cohan FM, Méric G (2017). Genomic plasticity and rapid host switching can promote the evolution of generalism: a case study in the zoonotic pathogen *Campylobacter*. Sci Rep.

[38] Bennett PM, Woodford Neil, Johnson Alan P (2004). Genome plasticity. Genomics, Proteomics, and Clinical Bacteriology Methods in Molecular Biology^TM^.

[39] Samarth DP, Kwon YM (2020). Horizontal genetic exchange of chromosomally encoded markers between *Campylobacter jejuni* cells. PLoS One..

[40] Sheppard SK, Cheng L, Méric G, Haan CPA, Llarena A, Marttinen P (2014). Cryptic ecology among host generalist *Campylobacter jejuni* in domestic animals. Mol Ecol.

[41] Baumler A, Fang FC (2013). Host specificity of bacterial pathogens. Cold Spring Harb Perspect Med.

[42] Nennig M, Llarena A-K, Herold M, Mossong J, Penny C, Losch S (2021). Investigating major recurring *Campylobacter jejuni* lineages in Luxembourg using four core or whole genome sequencing typing schemes. Front Cell Infect Microbiol.

[43] Sheppard SK, Colles F, Richardson J, Cody AJ, Elson R, Lawson A (2010). Host association of *Campylobacter* genotypes transcends geographic variation. Appl Environ Microbiol.

[44] Parker CT, Cooper KK, Schiaffino F, Miller WG, Huynh S, Gray HK (2021). Genomic characterization of *Campylobacter jejuni* adapted to the guinea pig (Cavia porcellus) host. Front Cell Infect Microbiol.

[45] Revez J, Schott T, Llarena A-K, Rossi M, Hänninen M-L (2013). Genetic heterogeneity of *Campylobacter jejuni* NCTC 11168 upon human infection. Infect Genet Evol.

[46] Thomas DK, Lone AG, Selinger LB, Taboada EN, Uwiera RRE, Abbott DW (2014). Comparative variation within the genome of *Campylobacter jejuni* NCTC 11168 in human and murine hosts. PLoS ONE.

[47] Kim J-S, Artymovich KA, Hall DF, Smith EJ, Fulton R, Bell J (2012). Passage of *Campylobacter jejuni* through the chicken reservoir or mice promotes phase variation in contingency genes Cj0045 and Cj0170 that strongly associates with colonization and disease in a mouse model. Microbiology.

[48] Jerome JP, Bell JA, Plovanich-Jones AE, Barrick JE, Brown CT, Mansfield LS (2011). Standing genetic variation in contingency loci drives the rapid adaptation of *Campylobacter jejuni* to a novel host. PLoS ONE.

[49] Golz JC, Epping L, Knüver M-T, Borowiak M, Hartkopf F, Deneke C (2020). Whole genome sequencing reveals extended natural transformation in *Campylobacter* impacting diagnostics and the pathogens adaptive potential. Sci Rep.

[50] Morley L, McNally A, Paszkiewicz K, Corander J, Méric G, Sheppard SK (2015). Gene loss and lineage-specific restriction-modification systems associated with niche differentiation in the *Campylobacter jejuni* sequence type 403 clonal complex. Appl Environ Microbiol.

[51] Alegbeleye OO, Sant’Ana AS (2020). Pathogen subtyping tools for risk assessment and management of produce-borne outbreaks. Curr Opin Food Sci.

[52] Ricke SC, Feye KM, Chaney WE, Shi Z, Pavlidis H, Yang Y (2019). Developments in rapid detection methods for the detection of foodborne *Campylobacter* in the United States. Front Microbiol.

[53] Kreling V, Falcone FH, Kehrenberg C, Hensel A (2020). *Campylobacter* sp.: Pathogenicity factors and prevention methods—new molecular targets for innovative antivirulence drugs?. Appl Microbiol Biotechnol.

[54] Ferrario C, Lugli GA, Ossiprandi MC, Turroni F, Milani C, Duranti S (2017). Next generation sequencing-based multigene panel for high throughput detection of food-borne pathogens. Int J Food Microbiol.

[55] Wassenaar TM, Newell DG (2000). Genotyping of *Campylobacter* spp. Appl Environ Microbiol.

[56] Maiden MCJ (2006). Multilocus sequence typing of bacteria. Annu Rev Microbiol.

[57] Gevers D, Cohan FM, Lawrence JG, Spratt BG, Coenye T, Feil EJ (2005). Re-evaluating prokaryotic species. Nat Rev Microbiol.

[58] Sheppard SK, Dallas JF, MacRae M, McCarthy ND, Sproston EL, Gormley FJ (2009). *Campylobacter* genotypes from food animals, environmental sources and clinical disease in Scotland 2005/6. Int J Food Microbiol.

[59] Pascoe B, Méric G, Yahara K, Wimalarathna H, Murray S, Hitchings MD (2017). Local genes for local bacteria: Evidence of allopatry in the genomes of transatlantic *Campylobacter* populations. Mol Ecol.

[60] Mouftah SF, Pascoe B, Calland JK, Mourkas E, Tonkin N, Lefevre C (2022). Local accessory gene sharing among Egyptian *Campylobacter* potentially promotes the spread of antimicrobial resistance. Microb Genomics.

[61] Köser CU, Ellington MJ, Cartwright EJP, Gillespie SH, Brown NM, Farrington M (2012). Routine use of microbial whole genome sequencing in diagnostic and public health microbiology. PLoS Pathog..

[62] Van Goethem N, Descamps T, Devleesschauwer B, Roosens NHC, Boon NAM, Van Oyen H (2019). Status and potential of bacterial genomics for public health practice: a scoping review. Implement Sci.

[63] Salipante SJ, SenGupta DJ, Cummings LA, Land TA, Hoogestraat DR, Cookson BT (2015). Application of whole-genome sequencing for bacterial strain typing in molecular epidemiology. J Clin Microbiol.

[64] Baker S, Thomson N, Weill F-X, Holt KE (2018). Genomic insights into the emergence and spread of antimicrobial-resistant bacterial pathogens. Science.

[65] Didelot X, Bowden R, Wilson DJ, Peto TEA, Crook DW (2012). Transforming clinical microbiology with bacterial genome sequencing. Nat Rev Genet.

[66] Revez J, Zhang J, Schott T, Kivistö R, Rossi M, Hänninen ML (2014). Genomic variation between *Campylobacter jejuni* isolates associated with milk-borne-disease outbreaks. J Clin Microbiol.

[67] Cody AJ, McCarthy ND, Jansen van Rensburg M, Isinkaye T, Bentley SD, Parkhill J (2013). Real-time genomic epidemiological evaluation of human *Campylobacter* isolates by use of whole-genome multilocus sequence typing. J Clin Microbiol.

[68] Fernandes AM, Balasegaram S, Willis C, Wimalarathna HML, Maiden MC, McCarthy ND (2015). Partial failure of milk pasteurization as a risk for the Transmission of *Campylobacter* from cattle to humans. Clin Infect Dis.

[69] Lahti E, Löfdahl M, Ågren J, Hansson I, Olsson EE (2017). Confirmation of a campylobacteriosis outbreak associated with chicken liver pâté using PFGE and WGS. Zoonoses Public Health.

[70] Clark CG, Berry C, Walker M, Petkau A, Barker DOR, Guan C (2016). Genomic insights from whole genome sequencing of four clonal outbreak *Campylobacter jejuni* assessed within the global *C. jejuni* population. BMC Genomics.

[71] Mughini-Gras L, Kooh P, Fravalo P, Augustin J-C, Guillier L, David J (2019). Critical orientation in the jungle of currently available methods and types of data for source attribution of foodborne diseases. Front Microbiol.

[72] Mullner P, Spencer SEF, Wilson DJ, Jones G, Noble AD, Midwinter AC (2009). Assigning the source of human campylobacteriosis in New Zealand: a comparative genetic and epidemiological approach. Infect Genet Evol.

[73] Thépault A, Méric G, Rivoal K, Pascoe B, Mageiros L, Touzain F (2017). Genome-wide identification of host-segregating epidemiological markers for source attribution in *Campylobacter jejuni*. Appl Environ Microbiol.

[74] Sheppard SK, Dallas JF, Strachan NJC, MacRae M, McCarthy ND, Wilson DJ (2009). *Campylobacter* genotyping to determine the source of human infection. Clin Infect Dis.

[75] Saber MM, Shapiro BJ (2020). Benchmarking bacterial genome-wide association study methods using simulated genomes and phenotypes. Microb Genom.

[76] Wilson DJ, Gabriel E, Leatherbarrow AJH, Cheesbrough J, Gee S, Bolton E (2008). Tracing the source of campylobacteriosis. PLoS Genet.

[77] McCarthy ND, Colles FM, Dingle KE, Bagnall MC, Manning G, Maiden MCJ (2007). Host-associated genetic import in *Campylobacter jejuni*. Emerg Infect Dis.

[78] Berthenet E, Thépault A, Chemaly M, Rivoal K, Ducournau A, Buissonnière A (2019). Source attribution of *Campylobacter jejuni* shows variable importance of chicken and ruminants reservoirs in non-invasive and invasive French clinical isolates. Sci Rep.

[79] Saif NA, Cobo-Díaz JF, Elserafy M, El-Shiekh I, Álvarez-Ordóñez A, Mouftah SF (2022). A pilot study revealing host-associated genetic signatures for source attribution of sporadic *Campylobacter jejuni* infection in Egypt. Transbound Emerg Dis.

[80] Clark CG, Taboada E, Grant CCR, Blakeston C, Pollari F, Marshall B (2012). Comparison of molecular typing methods useful for detecting clusters of *Campylobacter jejuni* and C. *coli* isolates through routine surveillance. J Clin Microbiol.

[81] Pritchard JK, Stephens M, Donnelly P (2000). Inference of population structure using multilocus genotype data. Genetics.

[82] Blaser MJ, Engberg J. 2008 Clinical Aspects of *Campylobacter jejuni* and *Campylobacter coli* Infections. In: *Campylobacter*. ASM Press: Washington. 97–121. https://onlinelibrary.wiley.com/doi/abs/10.1128/9781555815554.ch6

[83] Wieczorek K, Osek J (2013). Antimicrobial resistance mechanisms among *Campylobacter*. Biomed Res Int.

[84] Elhadidy M, Ali MM, El-Shibiny A, Miller WG, Elkhatib WF, Botteldoorn N (2020). Antimicrobial resistance patterns and molecular resistance markers of *Campylobacter jejuni* isolates from human diarrheal cases. PLoS ONE.

[85] Kittl S, Heckel G, Korczak BM, Kuhnert P (2013). Source attribution of human *Campylobacter* isolates by MLST and Fla-typing and association of genotypes with quinolone resistance. PLoS One..

[86] Kovač J, Čadež N, Stessl B, Stingl K, Gruntar I, Ocepek M (2015). High genetic similarity of ciprofloxacin-resistant *Campylobacter jejuni* in central Europe. Front Microbiol.

[87] Klein-Jöbstl D, Sofka D, Iwersen M, Drillich M, Hilbert F (2016). Multilocus sequence typing and antimicrobial resistance of *Campylobacter jejuni* isolated from dairy calves in Austria. Front Microbiol.

[88] Elhadidy M, Miller WG, Arguello H, Álvarez-Ordóñez A, Duarte A, Dierick K (2018). Genetic basis and clonal population structure of antibiotic resistance in C*ampylobacter jejuni* isolated from broiler carcasses in Belgium. Front Microbiol.

[89] Manyi-Loh C, Mamphweli S, Meyer E, Okoh A (2018). Antibiotic use in agriculture and its consequential resistance in environmental sources: potential public health implications. Molecules.

[90] Aksomaitiene J, Ramonaite S, Tamuleviciene E, Novoslavskij A, Alter T, Malakauskas M (2019). Overlap of antibiotic resistant *Campylobacter jejuni* MLST genotypes isolated from humans, broiler products, dairy cattle and wild birds in Lithuania. Front Microbiol.

[91] Karikari AB, Obiri-Danso K, Frimpong EH, Krogfelt KA (2017). Antibiotic resistance of *Campylobacter* recovered from faeces and carcasses of healthy livestock. Biomed Res Int.

[92] Karama M, Kambuyi K, Cenci-Goga BT, Malahlela M, Jonker A, He C (2020). Occurrence and antimicrobial resistance profiles of Campylobacter jejuni, Campylobacter coli, and Campylobacter upsaliensis in beef cattle on cow–calf operations in South Africa. Foodborne Pathog Dis.

[93] Mourkas E, Florez-Cuadrado D, Pascoe B, Calland JK, Bayliss SC, Mageiros L (2019). Gene pool transmission of multidrug resistance among *Campylobacter* from livestock, sewage and human disease. Environ Microbiol.

[94] Cobo-Díaz JF, González del Río P, Álvarez-Ordóñez A (2021). Whole resistome analysis in *Campylobacter jejuni* and *C. coli* genomes available in public repositories. Front Microbiol.

[95] Hodges LM, Taboada EN, Koziol A, Mutschall S, Blais BW, Inglis GD (2021). Systematic evaluation of whole-genome sequencing based prediction of antimicrobial resistance in *Campylobacter jejuni* and *C. coli*. Front Microbiol.

[96] Zhao S, Tyson GH, Chen Y, Li C, Mukherjee S, Young S (2016). Whole-genome sequencing analysis accurately predicts antimicrobial resistance phenotypes in *Campylobacter* spp. Appl Environ Microbiol.

[97] Giacomelli M, Andrighetto C, Rossi F, Lombardi A, Rizzotti L, Martini M (2012). Molecular characterization and genotypic antimicrobial resistance analysis of *Campylobacter jeju*ni and *Campylobacter coli* isolated from broiler flocks in northern Italy. Avian Pathol.

[98] Bortolaia V, Kaas RS, Ruppe E, Roberts MC, Schwarz S, Cattoir V (2020). ResFinder 4.0 for predictions of phenotypes from genotypes. J Antimicrob Chemother.

[99] Gibson MK, Forsberg KJ, Dantas G (2015). Improved annotation of antibiotic resistance determinants reveals microbial resistomes cluster by ecology. ISME J.

[100] Gupta SK, Padmanabhan BR, Diene SM, Lopez-Rojas R, Kempf M, Landraud L (2014). ARG-ANNOT, a New Bioinformatic Tool To Discover Antibiotic Resistance Genes in Bacterial Genomes. Antimicrob Agents Chemother.

[101] Alcock BP, Raphenya AR, Lau TTY, Tsang KK, Bouchard M, Edalatmand A (2020). CARD 2020: antibiotic resistome surveillance with the comprehensive antibiotic resistance database. Nucleic Acids Res.

[102] Feldgarden M, Brover V, Gonzalez-Escalona N, Frye JG, Haendiges J, Haft DH (2021). AMRFinderPlus and the Reference Gene Catalog facilitate examination of the genomic links among antimicrobial resistance, stress response, and virulence. Sci Rep.

[103] Hendriksen RS, Bortolaia V, Tate H, Tyson GH, Aarestrup FM, McDermott PF (2019). Using genomics to track global antimicrobial resistance. Front public Heal.

[104] Yahara K, Méric G, Taylor AJ, de Vries SPW, Murray S, Pascoe B (2017). Genome-wide association of functional traits linked with *Campylobacter jejuni* survival from farm to fork. Environ Microbiol.

[105] San JE, Baichoo S, Kanzi A, Moosa Y, Lessells R, Fonseca V (2020). Current affairs of microbial genome-wide association studies: Approaches, bottlenecks and analytical pitfalls. Front Microbiol.

[106] Brynildsrud O, Bohlin J, Scheffer L, Eldholm V (2016). Rapid scoring of genes in microbial pan-genome-wide association studies with Scoary. Genome Biol.

[107] Collins C, Didelot X (2018). A phylogenetic method to perform genome-wide association studies in microbes that accounts for population structure and recombination. PLOS Comput Biol..

[108] Earle SG, Wu C-H, Charlesworth J, Stoesser N, Gordon NC, Walker TM (2016). Identifying lineage effects when controlling for population structure improves power in bacterial association studies. Nat Microbiol.

[109] Lees JA, Galardini M, Bentley SD, Weiser JN, Corander J (2018). pyseer: a comprehensive tool for microbial pangenome-wide association studies. Bioinformatics.

[110] Sheppard SK, Didelot X, Meric G, Torralbo A, Jolley KA, Kelly DJ (2013). Genome-wide association study identifies vitamin B5 biosynthesis as a host specificity factor in *Campylobacter*. Proc Natl Acad Sci USA.

[111] Buchanan CJ, Webb AL, Mutschall SK, Kruczkiewicz P, Barker DOR, Hetman BM (2017). A genome-wide association study to identify diagnostic markers for human pathogenic *Campylobacter jejuni* strains. Front Microbiol.

[112] Méric G, McNally A, Pessia A, Mourkas E, Pascoe B, Mageiros L (2018). Convergent amino acid signatures in polyphyletic *Campylobacter jejuni* subpopulations suggest human niche tropism. Genome Biol Evol.

